# Dengue virus transmission in Italy: historical trends up to 2023 and a data repository into the future

**DOI:** 10.1038/s41597-024-04162-7

**Published:** 2024-12-05

**Authors:** Francesco Branda, Taishi Nakase, Antonello Maruotti, Fabio Scarpa, Alessandra Ciccozzi, Chiara Romano, Simone Peletto, Ana Maria Bispo de Filippis, Luiz Carlos Junior Alcantara, Alessandro Marcello, Massimo Ciccozzi, José Lourenço, Marta Giovanetti

**Affiliations:** 1 Unit of Medical Statistics and Molecular Epidemiology, Università Campus Bio-Medico di Roma, Rome, Italy; 2 Department of Epidemiology & Population Health, Stanford University School of Medicine, Stanford, CA USA; 3 Department GEPLI, Libera Università Ss Maria Assunta, 00193 Rome, Italy; 4 Department of Biomedical Sciences, University of Sassari, 07100 Sassari, Italy; 5 Istituto Zooprofilattico Sperimentale del Piemonte, Liguria e Valle d’Aosta, Turin, Italy; 6 Laboratório de Flavivírus, Instituto Oswaldo Cruz, Fundação Oswaldo Cruz, Rio de Janeiro, Brazil; 7 Instituto René Rachou, Fundação Oswaldo Cruz, Minas Gerais, Brazil; 8 The Laboratory of Molecular Virology, International Centre for Genetic Engineering and Biotechnology (ICGEB), 34149 Trieste, Italy; 9 Universidade Católica Portuguesa, Biomedical Research Center, Lisboa, Portugal; 10 Climate Amplified Diseases And Epidemics (CLIMADE), Europe, Lisboa, Portugal; 11 Sciences and Technologies for Sustainable Development and One Health, Università Campus Bio-Medico di Roma, Rome, Italy; 12 Oswaldo Cruz Institute, Oswaldo Cruz Foundation, Rio de Janeiro, Brazil

**Keywords:** Infectious diseases, Databases

## Abstract

Dengue virus circulation is on the rise globally, with increased epidemic activity in previously unaffected countries, including within Europe. In 2023, global dengue activity peaked, and Italy reported the highest number of dengue cases and local chains of transmission to date. By curating several sources of information, we introduce a novel data repository focused on dengue reporting in Italy. We integrate data from such a repository with other geographic, genomic and climatic spatiotemporal data to present an overview of transmission patterns of the past eight years related to circulating viral lineages, geographic distribution, hotspots of reporting, and the theoretical contribution of local climate. The novel data repository can contribute to a better understanding of an evolving epidemiological scenario in Italy, with the potential to inform reassessment and planning of adequate national and European public health strategies to manage the emergence of the dengue virus.

## Background & Summary

Dengue fever is a mosquito-borne viral infection caused by the dengue virus (DENV) of the *Flaviviridae* family, whose population structure is genetically (and antigenically) structured into four serotypes (DENV 1–4). Common symptoms include high fever, intense headaches, and significant muscle pain^1^. Transmission primarily occurs through the bites of *Aedes spp*. infected mosquitoes^1^. In recent years, there has been a significant global increase in incidence, leading to impacts on and concerns for public health systems. One of the primary drivers of this surge is climate change^2,3^. Ongoing climatic changes, including extreme temperature fluctuations and altered rainfall patterns, have not only contributed to the increased incidence but also facilitated geographic spread, notably towards higher altitudes^2^. Historically, Europe has mostly experienced imported cases of DENV, with sporadic autochthonous transmission (www.ecdc.europa.eu/en/dengue-monthly). However, autochthonous cases have been surging in the past five years, with significant reports from Spain^4^, Croatia^5^, France^6^ and Italy^7–9^. In 2023, Italy reported its highest ever number of locally transmitted DENV (www.epicentro.iss.it/febbre-dengue/aggiornamenti). The year was exceptional, not just due to the total number of reported cases, but also for the uncommon co-occurrence of a few, spatially disjointed autochthonous transmission chains of more than one DENV serotype.

Focusing on Italy, we introduce a novel, open-access spatiotemporal data repository that already includes 8 years of historical data on DENV infection reports (starting January 2015) and that will be updated into the future. The repository is intended to improve accessibility to information by the research, education and policy-making communities, thereby supporting the generation of knowledge and awareness related to the emergence of DENV in the country. When integrated with other diverse sources of information, e.g. geographic, genetic and climate-based, this dataset has the potential to contribute to a better understanding of the historical and real-time spatiotemporal dynamics and possible drivers of dengue activity in Italy. In this paper we explore such potential by exemplifying complementary data-driven approaches. For example, while the data records in the proposed dataset evidence the co-occurrence of DENV1 and DENV3 serotypes during the exceptional year of 2023, complementary phylogenetic analyses were able to identify the specific circulating genotypes (DENV1 genotype V, DENV3 genotype III) and infer their potential spatial origin into Italy. It is also described that the recent past climate-based suitability for DENV is lower than in endemic countries, which matches and partially justifies an historical lack of large autochthonous outbreaks or endemicity in the country. Critically, however, it is also shown that recent increases in temperature are particularly affecting regions of the country implicated in the emergence and increased epidemic activity of not just DENV, but also the Usutu and West Nile viruses. In general, it is suggested that the historical inter-year high, spatiotemporal variability in reported dengue in Italy is likely a result of a combination of local climatic conditions and broader transmission trends in endemic countries, particularly in South America.

The information summarized in this paper highlights the complexity of the emerging epidemiological scenario in Italy. There remains the need for a genomic surveillance network to better characterize the properties of viral introductions that result in local chains of transmission versus those that do not, and to critically reconstruct local chains of transmission to unfold how local dissemination takes place. A shift from passive to active surveillance would improve responsiveness to short-term transmission chains similar to those experienced during 2023. In parallel, active surveillance strategies should take into account factors such as the spatiotemporal variation of climate suitable for local transmission, mosquito-abundance spatial hotspots and seasonal human mobility from endemic countries. Together, this could help ensure a comprehensive approach to monitoring and containing DENV introductions and arboviruses more generally.

## Methods

The primary data source comes from a web dashboard by the Istituto Superiore di Sanità (ISS), which, in collaboration with the Ministry of Health, periodically publishes Surveillance and Response Plans to ensure early detection of cases and to minimize spread. A full description of the “National Arbovirus Prevention, Surveillance and Response Plan (NAP) 2020–2025” is available at www.salute.gov.it/imgs/C_17_pubblicazioni_2947_allegato.pdf.

**Fig. 1:**
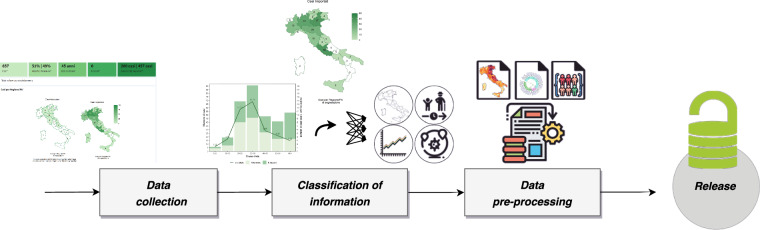
Schematic overview of the key steps involved in creating the open-access dataset.

Figure 1 summarizes the methodology developed to extract information and to construct the dataset. Specifically, during the “Data collection” step, several pieces of information were extracted from the dashboard of the EpiCentre website (www.epicentro.iss.it/arbovirosi/dashboard). After extraction, in the “Classification of information” step, information was structured according to the dashboard published by the ISS, which consists of the following sections: (i) a section presenting the incidence and distribution of dengue cases, including the total number of cases by gender and type of infection (autochthonous and imported), the total number of deaths, and the mean age of cases; (ii) a section reporting cases by region; (iii) a section reporting cases and incidence by gender and age group; (iv) a section providing information on the location of exposure of imported dengue cases; and (v) a final section presenting the timeline of confirmed cases. To ensure the consistency and enrichment of the dataset, a “Data pre-processing” step was necessary. Specifically, geographical information according to ISTAT nomenclature was added, including longitude and latitude. The weekly cases were calculated by determining the differences between the cumulative cases reported (‘total_cases’) in the current monitoring week. Additionally, raw data was extracted on the number of reported cases by gender and type of infection directly from the histograms in sections (iii) and (v). The most significant challenge was extracting information from the dashboard, as the data is, by default, presented in image format. Additionally, retrieving the historical data proved to be particularly complex because each week’s data replaced the previous week’s information. To address these challenges, we used the WebPlotDigitizer tool (www.apps.automeris.io/wpd) to extract data from the images with a high degree of precision. To reconstruct the history of the dashboard data, we used the Wayback Machine (www.wayback-api.archive.org), an online service that archives versions of specific websites and allows users to access them over time.

## Data Records

**Fig. 2:**
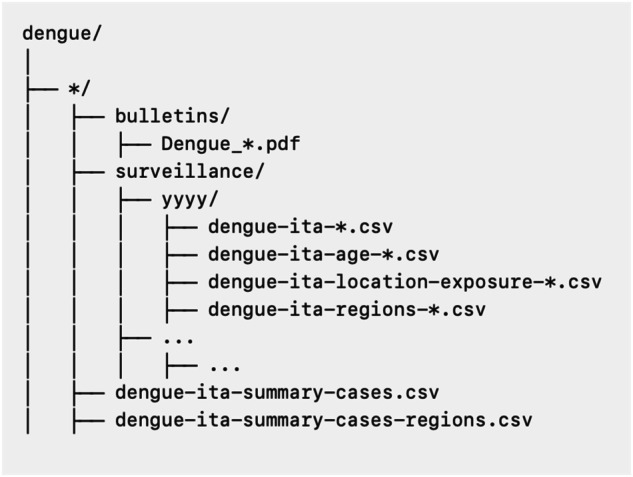
Schematic structure of the dataset.

The dataset, available at www.zenodo.org/records/13379740^10^ was structured as illustrated in Fig. 2. In summary, it is organized into two main folders: (i) a subfolder called “bulletins”, which includes all the PDF bulletins published by the ISS, and (ii) a subfolder called “surveillance”, which details national and regional trends in DENV cases. A detailed synopsis of the file structure is provided below.

### Bulletins

This folder contains PDF files reporting data on the epidemiological surveillance of DENV. Each bulletin is named “Dengue_yyyy.pdf,” where “yyyy” represents the year of publication. These bulletins include an annual summary of the dengue situation in Italy.

### Surveillance

This folder contains aggregated national and regional data for weekly DENV cases. These data are organized in comma-separated values (.csv) files, with each file corresponding to a monitoring year, as indicated by the “yyyy” designation at the end of the filename. Tables 1 and 2 describe the structure of the files named “dengue-ita-yyyy.csv” and “dengue-ita-regions-yyyy.csv”. These files are designed to collect detailed information, for example, on the distribution of cases by gender, the average age of positive cases, and the cumulative number of autochthonous and imported cases, further disaggregated by Italian region.

**Table 1:** Structure of the file “dengue-ita-yyyy.csv”.

Variable	Description	Format
data	Week reference date	yyyy-mm-dd
new_cases	New cases (“total_cases” current date - “total_cases” previous date) reported during the monitoring week	Numeric
total_cases	Cumulative number of cases reported since the beginning of the monitoring year	Numeric
new_deaths	New deaths (“total_deaths” current date - “total_deaths” previous date)	Numeric
total_deaths	Cumulative number of deaths reported since the beginning of the monitoring year	Numeric
perc_male_cases	Percentage of positive male cases reported since the beginning of the monitoring year	Numeric
perc_female_cases	Percentage of positive female cases reported since the beginning of the monitoring year	Numeric
median_age_cases	Average age of reported positive cases since start of monitoring	Numeric
autochthonous_cases	Cumulative number of autochthonous cases reported since the beginning of the monitoring year	Numeric
imported_cases	Cumulative number of imported cases reported since the beginning of the monitoring year	Numeric

**Table 2:** Structure of the file “dengue-ita-regions-yyyy.csv”.

Variable	Description	Format
data	Week reference date	yyyy-mm-dd
code_region	Region 2-digit code	Numeric
name_region	Region name	String
lat	Latitude of the region	Numeric
long	Longitude of the region	Numeric
new_cases	New cases (“total_cases” current date - “total_cases” previous date) reported during the monitoring week	Numeric
total_cases	Cumulative number of cases reported since the beginning of the monitoring year	Numeric

Table 3 outlines the structure of the file “dengue-ita-age-yyyy.csv”, which records new and cumulative cases reported for each age group, structured by default as 0–9, 10–19, 20–29, 30–39, 40–49, 50–59, 60 + years of age, along with the number of positive cases by gender and the percentage incidence of positive cases within each age group.

**Table 3:** Structure of the file “dengue-ita-age-yyyy.csv”.

Variable	Description	Format
data	Week reference date	yyyy-mm-dd
age	Age group (i.e. 0–9, 10–19, 20–29, 30–39, 40–49, 50–59, 60+)	String
new_cases	New cases (“total_cases” current date - “total_cases” previous date) reported during the monitoring week	Numeric
total_cases	Cumulative number of cases reported since the beginning of the monitoring year	Numeric
perc_male_cases	Percentage of positive male cases reported per age group during the monitoring week	Numeric
perc_female_cases	Percentage of positive female cases reported per age group during the monitoring week	Numeric
incidence_cases	Cumulative number of cases reported since the beginning of the monitoring year	Numeric

Table 4 describes the structure of the file “dengue-ita-location-exposure-yyyy.csv”, which is designed to track information on imported dengue cases in Italy. This file includes details such as the name of the location of exposure (i.e. where the infection occurred, if known) and the percentage of positive cases for each location.

**Table 4:** Structure of the file “dengue-ita-location-exposure-yyyy.csv”.

Variable	Description	Format
data	Week reference date	yyyy-mm-dd
location	Name of the place of exposure of imported DENV cases	String
percent_cases_week_exposure	Percentage of positive cases per exposure location reported during the monitoring week	Numeric

To provide a comprehensive overview of the available data at both national and regional levels, and to offer researchers and practitioners a concise and accessible version of the various surveillance data, two summary files were generated and made available, named “dengue-ita-summary-cases.csv” and “dengue-ita-summary-cases-regions.csv”. The structures of these files are detailed in Tables 5 and 6. The files are intended to help rapid comparison across regions, facilitating the identification of areas with lowest/highest burden, promoting a better understanding of the real-time epidemiological landscape, and hopefully supporting the adoption of targeted and timely interventions.

**Table 5:** Structure of the file “dengue-ita-summary-cases.csv”.

Variable	Description	Format
data	Week reference date	yyyy-mm-dd
month	Month of the monitoring year	String
total_cases	Cumulative number of cases reported since the beginning of the monitoring year	Numeric
autochthonous_cases	Cumulative number of autochthonous cases reported since the beginning of the monitoring year	Numeric
imported_cases	Cumulative number of imported cases reported since the beginning of the monitoring year	Numeric

**Table 6:** Structure of the file “dengue-ita-summary-cases-regions.csv”.

Variable	Description	Format
year	Monitoring year	yyyy
code_region	Region 2-digit code	Numeric
name_region	Region name	String
lat	Latitude of the region	Numeric
long	Longitude of the region	Numeric
total_cases	Cumulative number of cases reported since the beginning of the monitoring year	Numeric

## Technical Validation

The pipeline and specific steps taken to curate and validate data records composing the proposed dataset are described in detail in Methods. In this section, methods and strategies applied to existing data records up to 2023, demonstrating the validity and usefulness of the proposed dataset are presented. Using data available in the dataset, the historical spatiotemporal distribution of cases, serotypes and age distributions are described, and the risk and clustering of reports during 2023 are estimated. Phylogenetic inferences and climate-based transmission suitability are added to demonstrate possible data-driven venues to complete the information in the proposed dataset. All of the presented visual results were produced using the R (v4.3.1)^11^ package ggplot2^12^ with map boundaries obtained from the package geodata (v0.6-2)^12^.

We modeled reported counts per area $i$ as ${Y}_{i}$, ($i=1$ to n) using a Poisson distribution with mean ${E}_{i}\times {\theta }_{i}$, where ${E}_{i}$ is the expected counts and ${\theta }_{i}$ is the relative risk in area $i$. Then, the log risks are modeled with a sum of an intercept to model the overall disease risk level and random effects that account for extra-Poisson variability in the observed data^12^. Areas with relative risks ${\theta }_{i} > 1$ and ${\theta }_{i} < 1$ are areas with high and low risks, respectively. Areas with ${\theta }_{i}=1$ have the same risk as expected from the standard population. In this work, the model for disease mapping is expressed as:

$${Y}_{i} \sim {\rm{Poi}}({E}_{i}\times {\theta }_{i}),{\rm{i}}=1,\ldots ,{\rm{n}}$$

$${\log }({\theta }_{i})=\alpha +{u}_{i}+{v}_{i}$$

where $\alpha$ denotes the overall risk level, ${u}_{i}$ is a spatial structured random effect that models the spatial dependence between the relative risks, and ${v}_{i}$ is an unstructured exchangeable random effect that models uncorrelated noise.

We used Kulldorff’s scan statistics, applying moving windows with various shapes, such as circles or ellipses, and different sizes, running systematically over the entire study area. For each position and size of the window, the scan statistic calculates a probability index based on the ratio of the number of events observed within the window to the number of events expected under a null hypothesis of random distribution (i.e. the events are randomly distributed across the entire study area). The Kulldorff’s scan statistics then compares the actual data collected with this random distribution for each window, and calculates the likelihood ratio (LR) to determine whether the observed cluster is statistically significant or could be due to chance:

$${LR}=L({Observed},\psi )/L({Expected},\psi )$$

where $L({Observed},\psi )$ represents the likelihood of the observed data within the window, considering the parameters $\psi$ of the alternative hypothesis, and $L({Expected},\psi )$ represents the likelihood of the expected data within the window, also considering the parameters $\psi$ of the alternative hypothesis. An ${LR} > 1$ indicates that there are more observed cases within the window than expected. To determine the statistical significance of a cluster, LR is compared to a threshold value. This threshold is often represented as a -log(p-value). The p-value quantifies the probability of observing a cluster as extreme as, or more extreme than, the one identified under the null hypothesis that cases are randomly distributed. A lower p-value indicates a more significant cluster. More specifically, the -log(p-value) is compared to the critical values from a chi-squared distribution (χ^2^). If the log(p-value) is greater than or equal to the critical value from the chi-squared distribution at a specified significance level (often set at 0.05 or 0.01), the cluster is deemed statistically significant, and the null hypothesis of random distribution within the spatial window is rejected. Analyses were performed using R and SaTScan software (v10.1.2) (https://www.satscan.org/)^4^.

Available genome sequences from GenBank (DENV 1 n=7, DENV 3 n=3) were analyzed together with reference strains (n=95 for DENV1 and n=140 for DENV3, respectively) (Table S1). Sequences were aligned using MAFFT^13^ and manually refined in Aliview^14^ to eliminate anomalies. A GTR model, deemed the best fit by ModelFinder in IQ-TREE2^15^, was employed for the initial estimation of maximum likelihood phylogenetic trees, with robustness validated through 1000 bootstrap replicates. TempEst^16^ aided in detecting temporal signals, while BEAST facilitated the inference of time-scaled phylogenetic trees. A comprehensive model selection process, incorporating path-sampling and steppingstone methods, identified the uncorrelated relaxed molecular clock model, utilizing the SRD06 model and Bayesian Skygrid coalescent model, as optimal for Bayesian analysis^15,17^. Phylogeographic analyses were performed to map DENV’s spatial diffusion, using discrete sampling locations (countries) and an asymmetric model of location transition with BSSVS^18^. To ensure thoroughness, MCMC runs were performed in duplicate over 100 million iterations, achieving an effective sample size (ESS) of over 200. The final maximum clade trees, post 10% burn-in exclusion, were compiled using TreeAnnotator and displayed using ggtree^19^.

DENV climate-based transmission suitability was estimated as per Nakase *et al*.^3^ for each geographical pixel available in satellite climate data from Copernicus.eu^20^. This suitability measure (index P) measures the reproductive, transmission potential of a single adult female mosquito during its lifetime in a fully susceptible host population^21^. Since satellite data was only available up to September 2023, all outputs in the main text related to transmission suitability were summarized per year using climate data between January and September of each year. Long-term trends in local temperature were estimated using a linear regression model on each geographical pixel of the monthly satellite temperature time series from 1979 (January) to 2023 (September)^20^. Trends were summarized by estimated linear slopes (all had p-value < 0.0001).

### Epidemiological Characteristics and Dynamics of dengue in Italy up to 2023

**Fig. 3:**
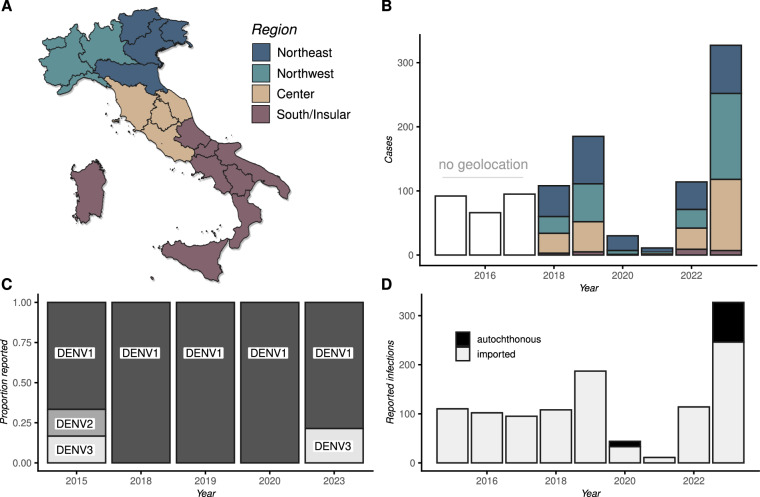
Spatiotemporal Dynamics of dengue in Italy. (**A)** Map of Italy with regions (black boundaries) and macroregions (color legend). (**B)** Time series of reported dengue cases. Reports between 2015–2017 had no geolocation, while those after 2018 are aggregated by macroregion. (**C)** Yearly count of available DENV genome sequences by serotype in the GenBank repository (January 2015 to November 20, 2023). Missing years had no genomes. (**D**) Number of reported infections over the years, categorized by whether transmission was concluded to be local (autochthonous, black) or foreign (imported, light grey).

Between 2015 and 20 November 2023, there have been a total of 1028 reported dengue cases nationally, including local and imported cases. Of these, 253 were reported between 2015 and 2017, for which there was no available geolocation (Fig. 3A). Between 2018 and 2023, a total of 775 cases were reported, with the Northeast, Northwest and Center macroregions each experiencing about 33% of cases (n=269, 256, 225, respectively), and the South/Insular experiencing only 3% of cases (n=25). The pandemic initial years of 2020 and 2021 presented the lowest reporting (Fig. 3B). While disruption to health services and awareness of infections beyond COVID-19 may have faltered during this period, when analyzing other data, we also uncovered that 2020 and 2021 presented some of the lowest climate-based transmission suitability over the years (explored later in the text, Fig. 6). During 2023, the year with highest ever reported cases (n=327, 31%), there were 75 cases in the Northeast, 134 in the Northwest, 111 in the Center and only 7 in the South/Insular macroregions (Fig. 3B). Among these, 82 were confirmed as autochthonous and attributable to four distinct chains of transmission within the regions of Lodi in the Northwest, and Latina, Rome (including metropolitan areas), Anzio, all in the Center macroregion.

Genomic data, derived from complete genome sequences available in the public repository (GenBank), reveal that three dengue serotypes (DENV1, DENV2, and DENV3) have circulated in Italy over the past eight years (Fig. 3C). Interestingly, DENV1 has been the predominant serotype reported in Italy, although the reasons for its dominance are not yet understood. In 2015, all three serotypes (DENV1, DENV2, and DENV3) were circulating. However, from 2018 to 2020, only DENV1 was detected in the database. Recently, in 2023, both DENV1 and DENV3 reappeared, suggesting a shift in serotype dynamics that warrants further investigation. This recent trend in Italy aligns with the global trends observed since early 2023, where there has been an increase in the circulation of DENV1 and DENV3, particularly across Latin American countries^1,22^. Reports have also been dominated by viral importations, with only the years of 2020 and 2023 having a significant contribution of cases resulting from autochthonous transmission (Fig. 1D).

**Fig. 4:**
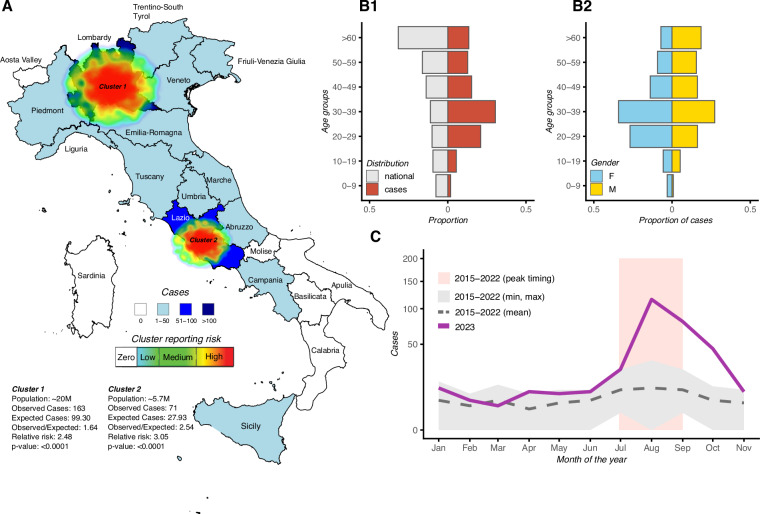
The 2023 Epidemiological Dynamics of dengue in Italy. (**A)** Identification of reporting hotspots across Italian regions in 2023, including a summary of key characteristics within the identified clusters. (**B1)** Breakdown of reported cases by age group. In red the proportion of cases per age group, and in grey the proportion of the national population per age group. (**B2)** Breakdown of reported cases by gender. In blue the proportion of cases reported as female per age group, and in yellow the proportion of cases reported as male per age group. For the period 2015–2022: grey dashed line is the monthly reporting mean; grey area marks the limits of the minimum and maximum reported cases over the months; pink area marks the typical time window of peak reporting. For 2023, the full purple line is the total cases per month. The y-axis is square root transformed for visualization.

By November 20, 2023, active dengue virus transmission was reported in 15 of Italy’s 21 regions. This wide geographical range prompted a nationwide movement, aimed at comprehensively assessing and identifying gaps in public health response and preparedness. Figure 4A delineates regions with high dengue incidence in 2023, categorized based on weekly case reports: Zero/Low includes regions like Abruzzo and Aosta Valley with up to five cases; Medium covers regions from Campania to Veneto with six to fifteen cases; and High represents Emilia Romagna, Lazio, Lombardy, and Piedmont, reporting over fifteen cases. Notably, Lombardy and Lazio represent 33% and 25% of the total cases, respectively, highlighting significant reporting concentrations. Two major clusters could be identified: the first extended across a diagonal axis within the Northwest and Northeast macroregions, from Piedmont to Emilia Romagna, covering over 20 million people; the second was located in Lazio, with a population of 5 million. The relative risk (RR) of dengue infection in these clusters was 2.48 in the first and 3.05 in the second.

The median age of individuals was 37 years, with a slight male predominance of 51.68% (Fig. 4B). In general, cases were more frequently reported among the 20–29 and 30–39 age groups, contrasting with the national age profile of the population (Fig. 4B1). Between genders, cases were also concentrated among the 20–29 and 30–39 age groups for females but were more uniformly distributed for males older than 20 years (Fig. 4B2). While differences in gender and age are typically reported for dengue in endemic countries, the specific reasons for described asymmetric exposures in Italy are unknown and should be the focus of future epidemiological studies. Compared to the monthly average trends in reported cases with the period 2015–2022, the year 2023 was different in both seasonal dynamics and size (Fig. 4C). Specifically, reports for 2023 were consistently higher than the historical average across the months (with the exception of March). Peak reporting in 2023 was in August (n=116) well within the historical time window of peak reporting but peaking much higher (ratio = 9.6) and only approaching average historical levels late in November.

### Phylodynamics Co-Circulating DENV1 and DENV3 in Italy up to 2023

**Fig. 5:**
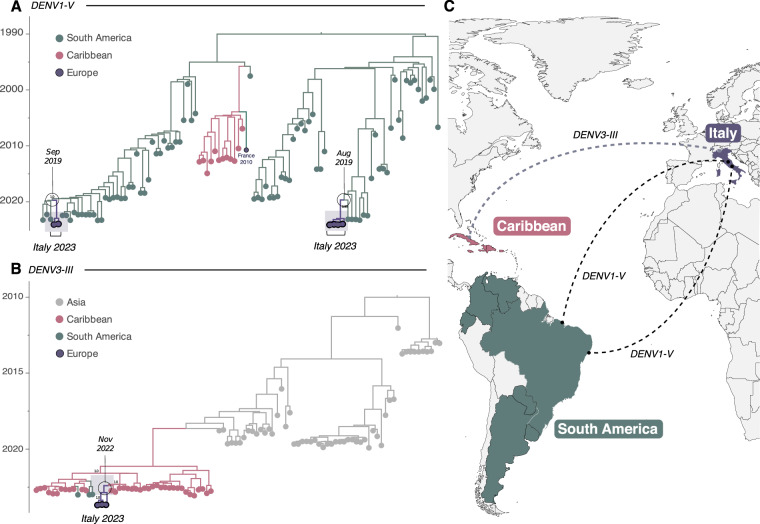
Phylodynamic analysis of DENV1-V and DENV3-III in Italy. (**A)** Time-scaled phylogeographic tree of DENV1-V (including all Italian strains currently available n = 7 plus n = 95 GenBank sequences). Colors represent different sampling locations. (**B)** Time-scaled phylogeographic tree of DENV3-III (including all Italian strains currently available n = 3 plus n=140 GenBank sequences). This analysis also includes sequences recently isolated in Florida from returning travelers who had visited Cuba, which have been highlighted as Caribbean in origin. Colors represent different sampling locations; (**C)** Map displaying the countries involved, with virus transmission paths marked by counterclockwise lines colored according to the viral source. DENV1-V is shown with a black dashed line, and DENV3-III is represented by a light green dashed line.

All available partial and complete DENV genome sequences from Italy, primarily generated during the 2023 epidemic, were collated with DENV-1 and DENV-3 genomes from other countries, available in public repositories. Phylogenetic analysis revealed that the 2023 isolates belonged to DENV-1 genotype V (DENV1-V) and DENV3 genotype III (DENV3-III), which coincidentally were the predominant genotypes circulating in Latin American countries at the time^1,23^. Further analysis of DENV1-V indicated two major independent introductions into Italy, estimated to have occurred in early and late December 2022 (High Posterior Density interval ranging from July 24, 2022, to June 10, 2023) (Fig. 5A). Discrete phylogeographic reconstruction also suggested that South America, primarily represented by Brazilian sequences, was a central hub for multiple viral introductions into Italy (Fig. 5B). Introduction of DENV3-III was traced back to late November 2022 (High Posterior Density interval ranging from August 22, 2022, to June 28, 2023) (Fig. 5C), suggesting that the Caribbean (including Cuba), which saw a rise in DENV3-III cases since 2022^1^, might have been the source of the introduction into Italy (Fig. 5D). In the context of these results, it should be noted that the small number of genomes available for Italy limits definite conclusions, and as such the results presented here are amenable to change upon inclusion of further genomic data.

### Climate-Based Transmission Suitability of dengue in Italy up to 2023

**Fig. 6:**
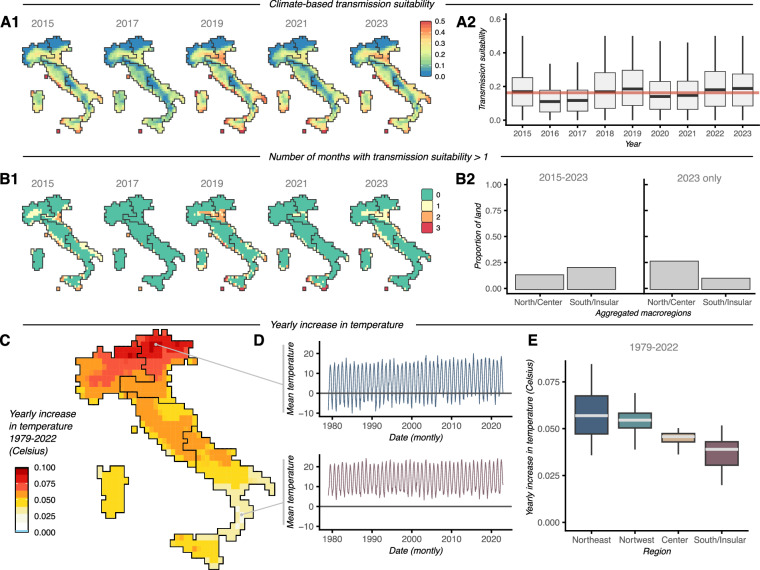
Climate-based Suitability of dengue in Italy. (**A1)** Yearly mean climate-based transmission suitability (index P) per geographical pixel of the climate satellite data; presented are a subset of years. (**A2)** Distributions of suitability values per year. The light red horizontal line marks the historical mean (2015–2023). (**B1)** Yearly number of months in which climate-based transmission suitability (index P) per geographical pixel of the climate satellite data was above 1; presented are a subset of years. (**B2)** Proportion of land (of geographical pixels available in climate data) that present suitability above 1 for at least 1 month of the year. Presented are the historical period (2015–2023) and 2023 only. North/Center is the aggregation of the Northwest, Northeast, and Center macroregions. (**C)** Map of Italy with estimates of rate of increase (linear slope) in mean yearly temperature (color legend). Black boundaries are macroregions. (**D)** Time series of mean monthly temperature (1979–2022) for two geolocations as marked on panel D. Line coloring follows the corresponding macroregion. (**E)** Distributions of rates of increase (linear slopes) for each of the macroregions (as presented in panel D).

Using climate satellite data, the DENV theoretical transmission suitability for Italy was estimated between 2015 and 2023. Suitability was found to vary across space (Fig. 6A1) and time (Fig. 6A2), being generally much lower than that estimated for DENV endemic countries such as Brazil or the Dominican Republic (see e.g.^2,24^), potentially explaining why long-term epidemics or endemicity have not been reported. The far north and the latitude-longitude diagonal across the country, rich in high altitude land, consistently presented the lowest suitability. In contrast, the island of Sicily, the continental coasts, and some areas of the northern macroregions consistently presented intermediate-to-high suitability. With the exception of Sicily, these areas were involved in the exceptional emergence of chains of transmission during 2023 (Figs. 3 and 4).

Suitability of 1 theoretically marks the threshold above which a single infected female mosquito can transmit the virus to more than one host during their lifetime and is typically used as a proxy for high transmission potential. Most of the country was estimated to have an unsuitable climate for transmission, and in fact across the years only a few hotspots showed high transmission potential (suitability >1) for a few consecutive months of a year (Fig. 6B1). The exceptional year of 2023 presented transmission suitability only slightly above the historical average (Fig. 6B1), suggesting that the increase in reported local cases may not have been driven by particularly suitable climatic conditions, but rather by the success of the virus elsewhere (from where it ended being imported). Indeed, 2023 was the worst year on record for South America, specifically for Brazil. In contrast to historical trends but following the geographical distribution of reported autochthonous cases in 2023, suitability above 1 occurred mostly in the Northwest, Northeast, and Center macroregions. (Fig. 6B2).

Estimates of historical trends in local temperature showed a universal, yearly mean increase across Italy (Fig. 6C). The highest rates of increase were found in the Northeast macroregion (Fig. 6D), followed by the Northwest, Center and South/Insular macroregions (Fig. 6DE). The highest signatures within the Northern macroregions conspicuously matched areas where West Nile virus and Usutu virus have emerged as public health threats in recent years^22,25^.

## Usage Notes

Building on our team’s previous work on pathogens such as Avian Influenza^26^, West Nile virus^27^, and Ebola^28^, this proposed dataset is specifically designed to enhance accessibility for the research, education and policy-making communities. Through an open-access repository, our primary goal is to foster collaboration and engagement among specialists, enabling students, researchers, and the public to explore and analyze data, thereby deepening their understanding of the impact of this virus in Italy. Together with other publicly available data and related methodologies, it serves the primary goal of supporting the generation of knowledge, raising awareness and contributing to a more comprehensive public health response about the ongoing emergence of dengue in Italy. The peer-reviewed data presented in this paper corresponds to version 3 (V3) of the dataset, which has been deposited on Zenodo (http://www.zenodo.org/records/13379740)^10^. A live version of the dataset is also available on GitHub (www.github.com/fbranda/dengue). To ensure traceability and consistency of results, any future updates to the dataset will be versioned and indicated separately.

The dataset introduced presents a few limitations that should be noted. For example, it includes only DENV reports that have been published by the ISS. Updating the dataset thus depends largely on the national reporting strategy adopted by the country, as well as on the intensity of the epidemiological research and surveillance strategy. In addition, media coverage of diagnosed cases has a significant influence, which may vary depending on social dynamics and the dissemination of information about DENV. In general, we have identified three main, external limitations in the proposed dataset: (i) incompleteness of collected data, including lack of detailed information on reported cases, such as precise geographic data (e.g. provincial cases); (ii) limitations in data representativeness due to factors such as underreporting of cases; (iii) limitations related to the availability of virus genomic data that can complicate the precise tracking of transmission dynamics and identification of circulating viral variants, especially if data are limited or missing for certain time periods or regions.

## Data Availability

The vast majority of developed code was related to the generation of the presented visualizations (Figs. 3–6), which we do not make publicly available in its entirety. Instead, we focus on making publicly available on Zenodo the code developed specifically for data analyses, namely to estimate DENV reporting risk and clustering, as well as to estimate DENV climate-based suitability (10.5281/zenodo.13379740)^10^.
